# NF-κB and IRF7 Pathway Activation by Epstein-Barr Virus Latent Membrane Protein 1

**DOI:** 10.3390/v5061587

**Published:** 2013-06-21

**Authors:** Ina Ersing, Katharina Bernhardt, Benjamin E. Gewurz

**Affiliations:** Division of Infectious Disease, Brigham and Women’s Hospital and Harvard Medical School, 181 Longwood Avenue, Boston, MA 02115, USA; E-Mails: inaersing@fas.harvard.edu (I.E.); katharinabernhardt@fas.harvard.edu (K.B.)

**Keywords:** herpesvirus, apoptosis, cancer, innate immunity, transformation, integral membrane protein, signal transduction, proliferation, ubiquitin, lymphoma, oncogene

## Abstract

The principal Epstein-Barr virus (EBV) oncoprotein, Latent Membrane Protein 1 (LMP1), is expressed in most EBV-associated human malignancies. LMP1 mimics CD40 receptor signaling to provide infected cells with constitutive NF-κB, MAP kinase, IRF7, and PI3 kinase pathway stimulation. EBV-transformed B-cells are particularly dependent on constitutive NF-κB activity, and rapidly undergo apoptosis upon NF-κB blockade. Here, we review LMP1 function, with special attention to current understanding of the molecular mechanisms of LMP1-mediated NF-κB and IRF7 pathway activation. Recent advances include the elucidation of transmembrane motifs important for LMP1 trafficking and ligand-independent signaling, analysis of genome-wide LMP1 gene targets, and the identification of novel cell proteins that mediate LMP1 NF-κB and IRF7 pathway activation.

## 1. Introduction

Epstein-Barr virus (EBV) is a gamma-herpesvirus that infects >90% of people worldwide, is the etiologic agent of infectious mononucleosis, and is associated with multiple human malignancies. Upon primary infection, EBV initially infects and may replicate in oropharyngeal epithelial cells [1,2]. EBV gains access to the B-cell compartment, where it drives robust B-cell proliferation through expression of six EBV nuclear antigens, multiple non-coding RNAs, and two integral membrane proteins, LMP1 and LMP2A [3,4]. Infected cells then enter lymph node germinal centers, where EBV gene expression is down-modulated, presumably to limit immune-detection. EBV subsequently establishes persistent infection of the memory B-cell compartment, from which it periodically reactivates [4,5,6]. 

The principal viral oncoprotein, LMP1, transforms rodent fibroblasts [7]. Transgenic LMP1 expression in murine models promotes the development of B-cell lymphomas and carcinomas [8,9,10,11]. Similarly, LMP1 expression is detectable in multiple human malignancies, where it may play a causal role. In particular, with T-cell immune-suppression, LMP1 is frequently expressed in proliferating B-cells of patients with lymphoproliferative disorders. These include post-transplant lymphoproliferative disorders and lymphomas in HIV-infected people [12]. EBV and LMP1 are also increasingly detected in diffuse-large B-cell lymphomas of the elderly [13]. In both immune-compromised and immunocompetent hosts, EBV and LMP1 are frequently present in the malignant Reed-Sternberg cell of Hodgkin Lymphoma (HL) [4,14,15,16]. Indeed, despite the success of antiretroviral therapy for human immunodeficiency virus, the risk of HIV-associated EBV-positive Hodgkin lymphoma has not decreased [17]. In immunocompetent hosts, LMP1 is expressed in a subset of anaplastic nasopharyngeal carcinomas (NPC) [18,19,20]. NPC is one of the most prevalent EBV-associated malignancies, and has a striking 50-fold higher incidence rate in southern China than in the Western world [21]. LMP1 is frequently expressed in these malignancies [15]. While latent EBV infection is detectable in roughly 10% of gastric carcinomas worldwide, only a subset appear to express LMP1 [22]. 

As described in further detail below, LMP1 signals through two cytoplasmic domains. Both domains are necessary for efficient B-cell conversion to immortal lymphoblasts (LCLs) [23,24,25,26]. LMP1 induces cell survival and growth through ligand-independent activation of multiple cell pathways. These include nuclear factor-κB (NF-κB), mitogen-activated protein kinase (MAPK), interferon-regulatory factor 7 (IRF7), and phosphatidylinositol 3-kinase (PI3K) pathways [27,28,29]. 

NF-κB transcription factors (TFs) are comprised of the REL-homology domain proteins p50, p52, RelA (also called p65), RelB, and cREL [30]. NF-κB TFs control lymphoid cell proliferation, differentiation and survival, and are critically important regulators of normal and pathological innate and adaptive immune responses. Inhibitory IκB proteins sequester NF-κB TFs in the cytosol to tightly restrict basal activity. Upon activation, receptor cytoplasmic domains initiate signal transduction cascades, referred to as NF-κB pathways [31]. Two major NF-κB pathways are recognized, and are termed the canonical and non-canonical NF-κB pathways. The cytosolic phase of NF-κB pathways culminates in IκB degradation, which allows NF-κB TF nuclear translocation. Post-translational modifications further activate NF-κB TFs, optimizing their binding to DNA κB sites and transcriptional regulation. Negative feedback loops tightly control NF-κB activation and terminate signaling upon stimulus withdrawal. While physiologic NF-κB activation is essential for lymphocyte development and activation, hyperactive NF-κB signaling promotes inflammatory diseases and malignant transformation [30]. 

Interferon regulatory factor (IRF) 7 is a key regulator of Type 1 IFN production [32] and belongs to a family of 9 human IRF genes [33]. Interestingly, the IRF family underwent co-evolution with NF-κB [34]. Nucleic acid-sensing pathogen recognition receptors (PRRs), including Toll-like receptor (TLR) 2, 3, 7, 9, RIG-I, and IFI16, activate IRF7 in response to pathogenic nucleic acids [35]. Several nucleotide binding oligomerization domain-like receptors, in particular NOD2, also activate IRF7 [36]. IRF7 contributes to interferon-γ-induced target gene regulation [37]. 

This review will highlight current understanding of LMP1-mediated NF-κB and IRF7 pathway activation, with special attention to recent advances in the field.

## 2. LMP1 Structure

**Figure 1:**
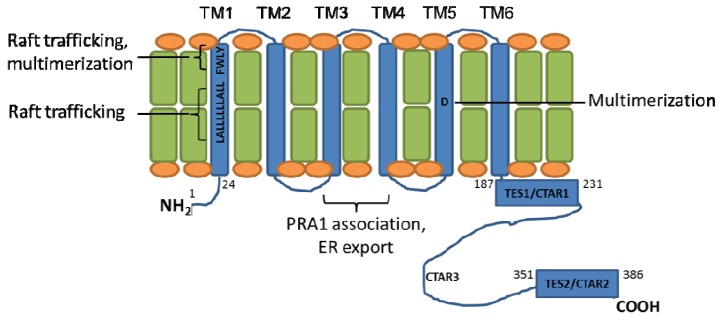
Latent Membrane Protein 1 (LMP1) transmembrane domain residues important for aggregation and signaling. The six LMP1 transmembrane (TM) domains (shown in blue) promote LMP1 multimerization and trafficking to cytoplasmic membrane (shown in green/orange) lipid raft signaling sites. Regions important for LMP1 signaling, aggregation, and trafficking are highlighted.

LMP1 contains 386 residues, comprised of a 24-residue N-terminal cytoplasmic tail domain, six transmembrane domains, and a 200 residue C-terminal tail (Figure 1). The LMP1 TM domains enable ligand-independent signaling from two LMP1 C-terminal tail domains. The C-Terminal Activating Region 1 (CTAR1) or Transformation Effector Site 1 (TES1) membrane proximal signaling domain (referred to TES1 hereafter) spans residues 187–231. Residues 351–386 comprise the membrane distal TES2/CTAR2 domain (referred to as TES2 hereafter). The LMP1 CTAR3 region resides between CTAR1 and CTAR2. While CTAR3 has been less well characterized, it binds to Janus kinase 3 (JAK3) and activates signal transducer and activator of transcription 1 (STAT1) [38]. However, EBV recombinants that lack the CTAR3 domain nonetheless transform B-cells with similar efficiency as wildtype strains. The resulting LCLs exhibit similar levels of tyrosine-phosphorylated JAK3, STAT3, and STAT5 as in LCLs established from wildtype virus [39,40]. More recently, the LMP1 CTAR3 region was found to interact with the small ubiquitin-like modifier (SUMO)-conjugating enzyme UBC9 and promote SUMOylation of IRF7 and other cell proteins (see IRF7 section below) [41,42]. CTAR3 stimulates SUMOylation of multiple cell proteins, and contributes to LMP1-induced effects on cell migration [42].

Other than a 5-residue tumor necrosis factor (TNF) receptor-associated factor (TRAF) binding motif, LMP1 does not exhibit amino acid similarity with CD40 (see below). Nonetheless, LMP1 and CD40 activate remarkably similar signal transduction cascades. TES1 and TES2 independently activate signal transduction pathways, and efficient long-term LCL outgrowth requires signaling from both domains [28]. 

## 3. LMP1 Transmembrane Domains Enable Constitutive LMP1 C-terminal Tail Signaling

The six LMP1 transmembrane domains (TM1-6) confer ligand-independent oligomerization, trafficking to membrane lipid rafts, and constitutive LMP1 C-terminal tail signaling [28]. Indeed, the cytoplasmic tails of CD40, FAS, TNFR1, and TNFR2 constitutively signal when fused to the LMP1 N-terminus and TM domains [43,44,45]. By contrast, when fused to the CD40 extracellular receptor and transmembrane domain, the LMP1 C-terminal tail signals only in response to CD40 ligand stimulation [46]. Transgenic mice that express this CD40/LMP1 fusion protein, but not endogenous CD40, still have normal B-cell development, activation, class-switch recombination, and germinal center formation [46]. However, LMP1 signaling, in this context, drives class-switch recombination to IgG1 independent of cytokines. How then do the LMP1 TM domains enable cytoplasmic tail signaling?

LMP1 TM residues important for LMP1 aggregation and trafficking have been identified through mutagenesis approaches (Figure 1). Deletion of LMP1 TM1-2 abrogates LMP1-mediated NF-κB activation, whereas deletion of LMP1 TM3-6 reduces LMP1-mediated NF-κB activation by ~60% [47]. Alanine mutagenesis of highly conserved human and rhesus EBV LMP1 TM1 residues identified a FWLY_38-41_ motif that is important for both LMP1 multimerization and lipid raft trafficking. Indeed, mutation of FWLY_38-41_ to AALA_38-41_ impairs LMP1 targeting to cholesterol and sphingolipid-rich lipid raft membrane microdomains, blocks TRAF3 recruitment, and markedly reduces LMP1-mediated NF-κB activation. Interestingly, FWLY_38-41_ also promotes intramolecular association between the LMP1 TM1-2 and TM3-6 domains. Additional intra-membrane associations further contribute to LMP1 aggregation and activation of LMP1 signaling [28,47]. 

LMP1 contains putative leucine heptad motifs in TM1 and TM6. Leucine heptads, which form leucine zipper-like structures, mediate protein-protein interactions [48]. Indeed, an important role for the TM1 leucine heptad is supported by mutagenesis studies. Alanine mutation of the TM1 leucine heptadimpairs LMP1-mediated NF-κB activation and B-cell transformation [49,50]. By contrast, mutation of the TM6 leucine heptad has minimal effects on LMP1 signaling [49]. The TM1 leucine heptad promotes LMP1 lipid rafts trafficking [50], where LMP1 recruits key signaling components. Likewise, LMP1 TM3-4 associate with the Golgi and late endosome protein prenylated rab acceptor 1 (PRA1). This association is important for LMP1 endoplasmic reticulum exit and initiation of C-terminal signal transduction [51].

Interestingly, LMP1 TM domains contain several charged and polar residues, which may play important roles in LMP1 aggregation. Some, but not all, LMP1 alleles contain an aspartic acid at residue 150. In the context of the B95.8 strain LMP1 TM5 domain, aspartic acid 150 (D150) may promote LMP1 multimerization. Alanine mutagenesis of D150 abrogates the ability of TM5 to homotrimerize in detergent micelles and reduces LMP1-mediated NF-κB activation in transfected cells, without substantially altering LMP1’s subcellular distribution [52]. Interestingly, the effect of D150 may be context dependent. In a recent analysis of LMP1 alleles from clinical EBV isolates, many LMP1s do not contain an aspartic acid at in TM5 [53]. EBV isolates with non-polar amino acids at position 150 nonetheless signal as well, or in some cases better, than B95.8 LMP1 in reporter assays. Interestingly, this increased NF-κB activity was nonetheless mapped to the clinical isolate LMP1 TM domains. Additional LMP1 TM amino acid substitutions (relative to B95.8 LMP1) may compensate for the absence of D150. 

## 4. LMP1-Mediated Canonical NF-κB Activation

**Figure 2:**
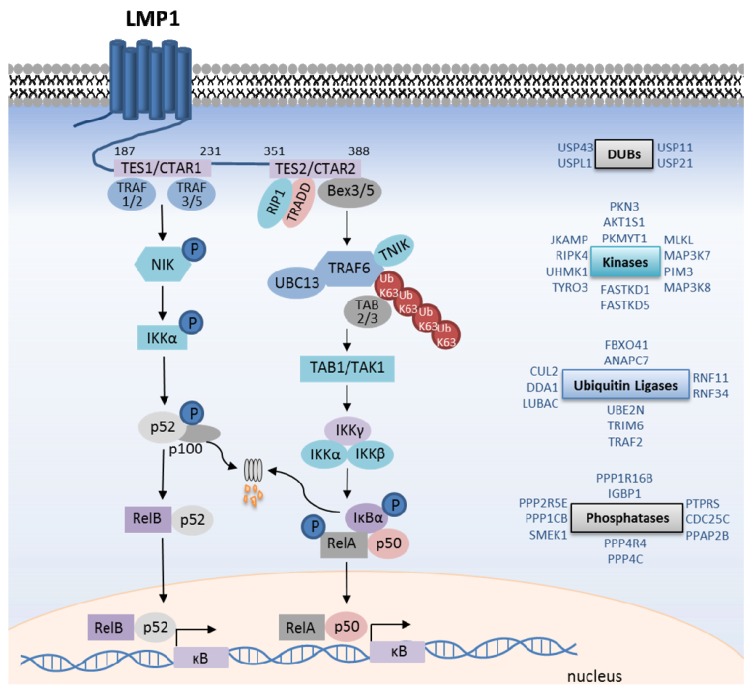
LMP1-mediated canonical and non-canonical NF-κB pathway activation. Shown are salient events in LMP1 TES1-mediated non-canonical NF-κB pathway activation, and TES2-mediated canonical pathway activation. Kinases are shown in purple, and ubiquitin ligases in blue. In addition, shown on the right, are additional kinases, phosphatases, ubiquitin ligases and deubiquitinating enzyme (DUB) siRNA screen hits that are important for TES2-mediated IκB kinase (IKK) complex activation. See text for full details.

TES2 predominantly activates the canonical NF-κB pathway (Figure 2). As described in more detail below, a hallmark of canonical NF-κB pathways is the activation of the IκB kinase (IKK) complex, comprised of the essential regulatory IKKγ (or NEMO) scaffold protein and the kinases IKKα and IKKβ. IKK phosphorylates the NF-κB inhibitor IκBα, which stimulates its rapid proteasomal degradation [30]. IκBα turnover allows canonical pathway NF-κB transcription factors, in particular the RelA/p50 heterodimer, to translocate to the nucleus.

Since LMP1 lacks intrinsic enzymatic activity, it recruits and activates enzymes. The K63-ubiquitin E3 ligase TRAF6 appears to be the first enzyme activated by TES2 [54,55,56,57]. While TRAF6 has been reported to bind to TES1 in murine B cells [58], we have been unable to detect a direct association between TES2 and TRAF6. How then does TES2 activate TRAF6? Multiple proteins have been suggested to bind directly to TES2, including RIP1 and TRADD [59]. TRADD knockout in DG75 B-cells abrogates TES2-mediated IKKβ recruitment and activation [60]. However, TRADD may not participate directly in TRAF6 activation, as TRADD deficiency does not impair LMP1-induced JNK activation, which is dependent on TRAF6 activation [60]. Furthermore, LMP1 may use TRADD in a cell-type specific fashion, as TRADD knockdown in 293 cells caused significant impairment of TNFα-mediated, but not TES2-mediated NF-κB activation [55,61]. Likewise, RIP1 may not be required for TES2-mediated NF-κB activation, as RIP1 knockdown in 293 cells and absence of detectable RIP1 protein in chemically mutagenized Jurkat T-cells do not impair TES2-mediated NF-κB activation [61,62].

Multiple adaptor proteins have been suggested to link LMP1 TES2 to TRAF6, including BS69 [63]. We found that the brain-expressed (BEX) family proteins BEX3 (also called nerve growth factor receptor associated protein 1, or NGFRAP1) and BEX5 associate with LMP1, and that BEX3/5 knockdown markedly impairs TES2-mediated IKK activation [61]. Interestingly, the p75 nerve growth factor (NGF) receptor, a member of the TNFα-receptor superfamily, binds multiple BEX family members [64], and BEX2 potentiates NGF-mediated NF-κB activation in breast cancer cells [65]. Recently, the TRAF2- and NCK-interacting kinase TNIK was identified as a novel TES2 interactor [66]. TNIK knockdown impairs LMP1-mediated IKK activation in 293 cells by nearly 50%, and diminishes LCL growth and survival. Interestingly, the N-terminal TNIK kinase domain is important for LMP1-mediated canonical NF-κB activation, whereas its C-terminal scaffold domain instead promotes JNK pathway activation [66]. 

LMP1 TES2 activates TRAF6 K63-ubiquitin ligase activity and promotes TRAF6 auto-K63-ubiquitination [54,55,56,58,67]. Indeed, TRAF6 knockdown strongly abrogates TES2-mediated NF-κB activation, and TRAF6 siRNAs were amongst the strongest TES2/NF-κB inhibitors in our genome-wide siRNA screen. Likewise, disruption of UBC13, TRAF6’s E2 ubiquitin ligase, impairs TES2-mediated NF-κB activation [56,61]. K63-ubiquitin chains recruit the homologous zinc-finger proteins TAB2 and TAB3, which in turn activate the kinase TAK1 (Figure 2). Further supporting an important role for K63-ubiquitin downstream of LMP1, combined TAB2/3 depletion impairs LMP1-mediated IKK activation [56]. Activated TAK1 phosphorylates downstream targets, in particular the IKK kinase activation loops. While TAK1 activity is obligatory for most canonical NF-κB pathways, TAK1 knockdown reduced TES2 NF-κB activation by only 40%–50% [61], and similar results have been observed in TAK1 deficient MEFs [68]. Thus, LMP1 may use additional kinase(s) in a partially-redundant manner with TAK1 [61]. Interestingly, our siRNA analysis indicates that several additional kinases are important for LMP1-mediated IKK activation (Figure 2). Further studies are required to determine whether any function at the level of TAK1. 

The recently discovered linear ubiquitin chain assembly complex (LUBAC) has increasingly been implicated in canonical pathway IKK activation, including by TNFα, interleukin 1-β (IL-1β), NOD2, and CD40 [69,70]. LUBAC is comprised of three components: the catalytic HOIP and HOIL-1L subunits, and the SHARPIN regulatory subunit. Interestingly, combined HOIP/HOIL-1L depletion impairs LMP1 TES2-mediated NF-κB activation by nearly 50% upstream of IKK complex activation [61]. By contrast, LUBAC depletion does not inhibit LMP1-mediated non-canonical NF-κB activation [71]. Open questions include how LMP1 signaling recruits and activates LUBAC to participate in downstream signaling, and the identification of cell LUBAC targets. While CD40 recruits HOIL-1L [72], we have not yet been able to detect direct association between LMP1 and LUBAC subunits. Multiple receptors induce LUBAC to attach linear ubiquitin chains to RIP1, NEMO and likely additional cell proteins, thereby promoting the assembly and stabilization of receptor-associated signaling complexes [73,74]. Identification of LUBAC substrates downstream of LMP1 remains to be elucidated. 

HOIL-1L deficiency causes a fatal inherited disorder with chronic autoinflammation, recurrent invasive bacterial infection, and muscular amylopectinosis [75]. IL-1β responses are impaired in fibroblasts but are hyper-responsive in mononuclear leukocytes from patients with HOIL-1L deficiency. Interestingly, EBV-transformed lymphoblastoid cells can be established from patient-derived B-cells, despite the presence of HOIL-1L loss-of-expression or loss-of-function mutations [75]. Curiously, CD40-mediated IKK phosphorylation, IκBα degradation, and NF-κB activation are markedly impaired in HOIL-1L-deficient LCLs. Since LMP1 mimics CD40 signaling, and since LMP1 is necessary for EBV-mediated B-cell transformation, how then can HOIL-1L deficient LCLs be derived? Perhaps HOIP plays a more important role downstream LMP1 than CD40, and residual LUBAC activity in HOIL-1L deficient LCLs is sufficient for LMP1 canonical pathway activation. Indeed, Sharpin and RNF31 knockdown more strongly impair LMP1 TES2/NF-κB than HOIL-1L knockdown [61]. Alternatively, perhaps LMP1-mediated non-canonical activity is sufficient to support EBV-mediated B-cell transformation. 

The IKK scaffold protein NEMO is critically important for LMP1-mediated canonical NF-κB activation, and siRNAs against NEMO were amongst the strongest LMP1 inhibitors detected in our genome-wide screen. NEMO binds linear and K63-ubiquitin chains, and through incompletely understood mechanisms, activates IKKα and IKKβ. Disruption of the NEMO linear ubiquitin binding domain (UBAN) abrogates LMP1-mediated canonical NF-κB activation [57]. By contrast, disruption of the NEMO K63-ubiquitin binding domain does not significantly impair TES2 NF-κB activation. Our systematic siRNA analysis implicated additional ubiquitin ligases and deubiquitinating enzymes in TES2-mediated IKK activation [61] (Figure 2). These may function in LMP1 trafficking, stability, or signaling to IKK, such as by regulating the stability of established TES2/NF-κB activation or inhibition pathway components.

Most canonical NF-κB pathways require IKKβ, but not IKKα activity. Key IKK targets include IκBα and RelA. IKK-mediated IκBα serine 32,36 phosphorylation stimulates its ubiquitination and proteasomal degradation, which then allows nuclear translocation of the RelA/p50 heterodimer (Figure 2). Interestingly, TES2 uses IKKα and IKKβ in partially redundant manners. While depletion of either kinase partially impairs TES2-mediated IκBα phosphorylation, degradation, RelA serine 536 phosphorylation, and reporter gene activation, combined IKKα/β depletion markedly blocks each of these, to a similar extent as NEMO depletion [61]. 

Following NF-κB transcription factor nuclear translocation, additional cellular mechanisms are likely to further regulate LMP1-mediated NF-κB activation. Indeed, knockdown of multiple putative nuclear proteins impair LMP1 mediated NF-κB reporter gene activation without affecting IKK activation [61]. For instance, multiple independent siRNAs against the little characterized zinc finger protein ZC3H13 impair NF-κB activation by TES2, as well as TNFα and IL-1β [76]. By contrast, multiple ZC3H-family members negatively regulate canonical NF-κB pathways, including ZC3H12a, whose knockout causes a lethal autoimmune syndrome in mice [76]. Further studies are required to determine how ZC3H13 instead promotes nuclear NF-κB activation. Interestingly, the closely related zinc finger protein ZC3H18 is important for TES2-mediated IKK activation [61].

TES2 up-regulates multiple well-characterized negative NF-κB regulators, including IκBα, A20, CYLD, and ABIN1 [77]. Indeed, these are amongst the earliest and most-robustly TES2-induced genes in HEK-293 cells [77]. Proteasomal degradation and exosome secretion also limit LMP1 cell abundance and therefore signal strength [78]. Indeed, LMP1 associates with the late endosome marker CD63, and a substantial fraction of LMP1 exits cells by exosome secretion from nasopharyngeal carcinoma and LCLs [79]. CD63 association is critical for LMP1 exosome secretion. Despite its important role in LMP1 signaling, lipid raft association appears not to be critical for LMP1 sorting into exosomes [80]. LMP1-containing exosomes, obtained from NPC or LCL cultures, induce LMP1 signal transduction pathway activation in neighboring cells [81].

Of note, LMP1 TES1 also activates canonical NF-κB through an incompletely characterized pathway. An LMP1 mutant truncated after the TES1 domain (LMP1 1-231) initiates primary B-cell growth transformation in tissue culture, and the resulting LCLs have similar nuclear NF-κB complexes as wild-type LCLs, including p50/RelA and p50/cREL heterodimers [25]. Likewise, we have observed that LMP1 1-231 activates canonical NF-κB activation in HEK-293 cells. TES1-mediated canonical NF-κB activity is blocked by NEMO knockdown or by over-expression of an IκBα super-repressor [71]. 

## 5. LMP1 Canonical NF-κB Gene Targets

LMP1 up-regulates and down-regulates a significant number of cell genes in both epithelial and B-cells [16,77,82,83,84]. Differences in chromatin accessibility between B-cells and epithelial cells may strongly configure the transcriptional landscape in response to LMP1 [85]. Unfortunately, direct comparisons of LMP1 B-cell *versus* epithelial cell transcriptional effects are limited by differences in experimental design and microarray platforms used in published studies. Nonetheless, in both cell types, important gene targets include NF-κB pathway components and feedback regulators, proteins important for cell cycle progression, blockade of apoptosis, immune-modulation, cytokines and cytokine receptors, and cell migration. In EBV-transformed lymphoblastoid cells, RelA binding is detectable at 58% of genes up-regulated by TES2 in HEK-293 cells [77]. 

While TES2 activates the p38, JNK, ERK and NF-κB pathways, canonical NF-κB activity is critical for TES2 target genes effects in HEK-293 cells. Indeed, whereas TES2 causes >2-fold changes in 1916 cell mRNAs, co-expression of an IκBα super-repressor together with TES2 decreases TES2 effects to only cell 5 mRNAs [77]. Of note, NF-κB inhibition has a strong, but less pronounced effect on LMP1 target gene regulation in BL41 Burkitt lymphoma cells [84].

Interestingly, an important role for LMP1-mediated canonical NF-κB activation in cell metabolism and glucose uptake has recently been elucidated [86]. Likewise, LMP1 up-regulates the expression of the cell microRNA miR-34a in a canonical NF-κB-dependent manner [87]. 

## 6. LMP1-Mediated Non-Canonical NF-κB Activation

In unstimulated cells, the ubiquitin ligases TRAF2, TRAF3, cIAP1 and cIAP2 target the kinase MAP3K14 (also called NF-κB inducible kinase, or NIK) for degradation and thereby suppress the non-canonical NF-κB pathway [88]. Thus, although NIK is constitutively made, its levels do not accumulate in cells in the absence of stimulation. TRAF3 serves as an adaptor that recruits the TRAF2/cIAP1/2 ubiquitin ligase complex, which then attaches degradative K48-linked ubiquitin chains to NIK. Human cell receptors, such as CD40 and the BAFF receptor, activate non-canonical NF-κB activity by disrupting the TRAF2/3/cIAP1/2 complex. Receptor activation causes TRAF2 to attach K63-ubiquitin chains to cIAP1 and cIAP2, which are then redirected to K48-ubiquitinate TRAF3 and stimulate its rapid degradation. In the absence of TRAF3, NIK is stabilized, and upon reaching a threshold concentration, presumably auto-activates its kinase activity [89]. NIK in turn phosphorylates IKKα, which then phosphorylates the p100 NF-κB transcription factor precursor (Figure 2). P100 phosphorylation stimulates proteasomal cleavage of its C-terminal IκB domain, generating the active p52 form. P52 then translocates to the nucleus as a homodimer, or as a heterodimer with other NF-κB transcription factors, in particular RelB [88].

LMP1 TES1 strongly activates the non-canonical NF-κB pathway, by an incompletely understood mechanism. To initiate signaling, the TES1 PQQAT_208_ motif recruits TRAFs 1, 2, 3, and 5 [90,91]. Mutation of this site to AQAAA_208_ abolishes TES1-mediated non-canonical activation. However, in contrast to signaling by CD40 and BAFF receptors, TES1 has not been observed to trigger TRAF3 degradation [92]. How then does TES1 activate the non-canonical pathway? Perhaps LMP1 sequesters sufficient TRAF3 away from TRAF2/cIAP1/2 complex to allow NIK to escape degradation. Indeed, the *Herpesvirus ateles* oncoprotein Tio activates non-canonical NF-kB by redistributing TRAF3 away from the cytosol in a ubiquitin-independent manner [93]. Alternatively, LMP1 may use TRAF3 to more directly activate non-canonical signaling by a unique mechanism [94]. Unfortunately, genetic analysis of TRAF2, TRAF3, and cIAP function downstream of LMP1 TES1 is complicated by high-level non-canonical NF-κB activity that results upon TRAF2 or TRAF3 depletion, even in the absence of stimulus. Though the precise mechanisms by which TES1 mediates NIK activation remain to be fully detailed, NIK has been established as a critical pathway component. Overexpression of a dominant-negative NIK mutant blocks TES1-mediated non-canonical activation in HEK-293 cells, and TES1 non-canonical NF-κB activation is blocked in MEFs that lack functional NIK [95,96,97]. The zinc finger protein ZFP91 promotes K63-ubiquitination of NIK and up-regulates NIK activity, perhaps by promoting NIK stability or potentiating its kinase activity [98]. ZFP91 knockdown also impairs CD40-mediated non-canonical NF-κB activation. Whether ZFP91 similarly functions downstream of LMP1 TES1 awaits further analysis. Likely through phosphorylation by NIK, LMP1 triggers IKKα activation, p100 phosphorylation, and p100 processing to p52 (Figure 2). P52 heterodimers, in particular p52/RelB, translocate to the nucleus to modulate target gene expression. Of note, p52/RelA complexes are also abundantly generated [96].

As with the LMP1 canonical NF-κB pathway, little is known about the nuclear phase of the LMP1 non-canonical NF-κB pathway, though additional regulatory mechanisms exist. Likewise, little is presently known about which genes are targeted by the LMP1-mediated canonical *versus* the non-canonical NF-κB pathways, the degree to which the two gene sets overlap, and the magnitude of canonical *vs.* non-canonical pathway effects on target gene regulation. 

## 7. LMP1/Atypical NF-κB Pathway Activation

LMP1 TES1 signaling induces epidermal growth factor receptor (EGFR) expression, even in murine embryonic fibroblasts that lack IKKα, IKKβ, or IKKγ. This activity does however require TRAFs 2, 3, and NIK, and culminates in the activation of a complex of p50 homodimer and Bcl-3 [99]. 

## 8. The LMP1/IRF7 Pathway

**Figure 3:**
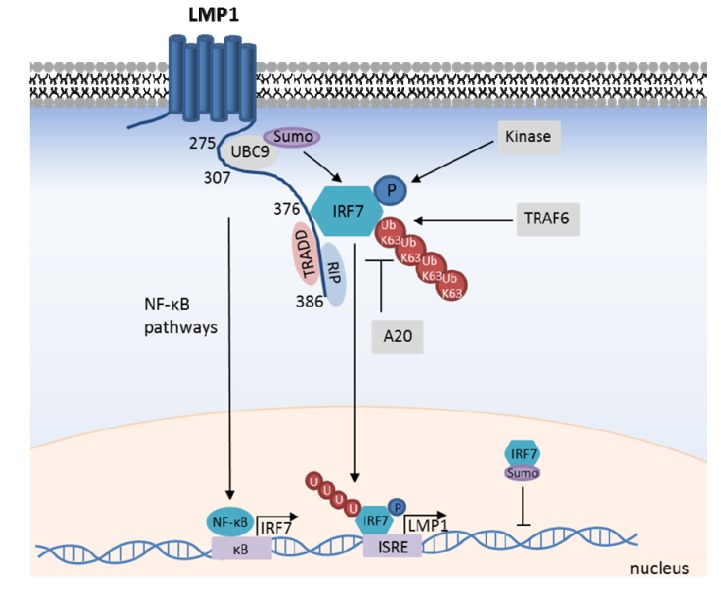
LMP1-mediated IRF7 activation. LMP1 recruits IRF7, promotes its TRAF6-mediated K63-ubiquitination, and C-terminal phosphorylation by as yet unidentified kinase(s). IRF7 translocates to the nucleus to activate target gene transcription, including LMP1 itself. IRF7 transcription is likewise up-regulated by LMP1-mediated NF-κB activation, which creates a positive feedback loop. IRF7 activity is negatively regulated by the DUB A20, and by LMP1-mediated recruitment of the SUMO ligase UBC9. IRF7 SUMOylation impairs its interaction with chromatin.

Studies of the EBV EBNA1 Q promoter originally led to the identification and cloning of IRF7, where IRF7 was found to bind to an interferon-stimulated response element (ISRE) and to promote type III latency [100]. LMP1 not only up-regulates IRF7 expression, but also activates IRF7, likely through a direct association with LMP1 [101,102,103]. Indeed, the LMP1 TES2 domain and IRF7 strongly interact by yeast 2-hybrid analysis, suggesting that LMP1 and IRF7 may directly interact [101,103,104] (though it remains possible that an adaptor protein present in yeast could facilitate the observed association between LMP1 and IRF7). Indeed, immunofluorescence analysis demonstrates substantial co-localization between LMP1 and IRF7 in human B-cell lines [102]. The LMP1/IRF7 association r LMP1 residues 379–386 [101,103,104]. Interestingly, while TRADD and RIP1 bind to a similar region of LMP1, they appear not to be required for LMP1-mediated IRF7 recruitment. Indeed, mutation of LMP1 residues YYD_386_ to ID abrogates TRADD and RIP1, but not IRF7 recruitment. The LMP1 ID mutant nonetheless does not activate the IRF7 pathway, consistent with important roles for TRADD and/or RIP1 in LMP1-mediated IRF7 activation [101,103,104]. While TRAF6 is important for LMP1-mediated IRF7 activation, TRAF2 and TRAF3 are dispensable [101,103,104]. Interestingly, LMP1 promotes TRAF6 K63-linked ubiquitination of three C-terminal IRF7 residues (positions 444, 446 and 452). Lysine to arginine mutation of these IRF7 residues abrogates IRF7 transactivation activity in response to either LMP1 or overexpression of an IRF7 kinase, IKKε. TRAF6-mediated IRF7 ubiquitination appears to be a prerequisite for its phosphorylation [104] (Figure 3). 

Though not yet formally demonstrated in the context of LMP1, C-terminal IRF7 phosphorylation generally promotes its nuclear translocation and target gene activation. For instance, PRR activation triggers phosphorylation of the inactive form of IRF7, enabling its subsequent nuclear translocation and subsequent transcription activation of target genes. Indeed, alanine mutation of IRF7 serines 477 and 499 abrogates its ability to transactivate target genes upon over-expression or in response to LMP1 [101,105]. The kinase(s) responsible for LMP1-mediated IRF7 phosphorylation have not yet been identified. Since IKKε, TBK1, IRAK1, and IKKα phosphorylate IRF7 in other contexts [35], it is likely that one or more of these kinases activate IRF7 downstream of LMP1. Studies of LMP1-mediated IRF7 phosphorylation in MEFs deficient for these kinases or RNAi approaches may identify the responsible kinase(s). Together with other co-activators, IRF7 can form transcription complexes that bind to target gene regulatory elements and activate transcription [106,107]. Additional components of the LMP1/IRF7 pathway remain to be discovered, and RNAi-mediated reverse genetic analysis of the pathway promises to yield additional insights into LMP1-mediated IRF7 activation.

IRF7 is predominantly expressed in lymphoid cells of the spleen, thymus and peripheral blood [108]. However, four NF-κB binding sites are present in the IRF7 gene promoter region, and LMP1 induces IRF7 expression in an NF-κB dependent manner [109,110]. Since IRF7 likewise up-regulates LMP1 expression, LMP1 and IRF7 participate in a positive-feedback circuit (Figure 3) [109,110,111]. 

Though elegant biochemical studies have established that LMP1 activates IRF7, the role of the LMP1/IRF7 pathway during EBV host infection remains to be fully characterized. Together with NF-κB, IRF7 may be responsible for the regulation of genes that contribute to cell growth and proliferation in EBV-transformed B-cells. Since both LMP1 and IRF7 have oncogenic properties, IRF7 may play an important role in EBV-associated malignancies. Indeed, IRF7 expression was frequently detected in LMP1-positive primary lymphomas of the human central nervous system, and the association between LMP1 and IRF7 expression was statistically significant. LMP1 and IRF7 co-expression demonstrate additive effects on the NIH 3T3 cell growth transformation [112]. Identification of the full suite of LMP1/IRF7 target genes would significantly enhance current understanding of IRF7’s role downstream of LMP1. Thus, RNA profiling and phenotypic analysis of IRF7-depleted EBV-transformed cells, as well as IRF7 ChIP-Seq analysis, may further elucidate IRF7’s role in EBV-associated malignancies. Likewise, despite robust IRF7 activation, LCLs do not produce substantial amounts of type I interferon [35]. Why IRF7 activation in this context does not lead to more substantial type I IFN induction during EBV latency awaits further studies.

Several negative regulators of the LMP1/IRF7 pathway have been identified. Perhaps attracted by robust IRF7 K63-ubiquitination, the deubiquitinating enzyme A20 is recruited to IRF7 and down-modulates its activity. A20 over-expression impairs LMP1-mediated IRF7 activation, whereas A20 knockdown enhances it [113]. This inhibitory effect requires the A20 deubiquitinase domain, but not its E3 ubiquitin ligase domain. Similarly, SUMOylation down-modulates IRF7 activity. LMP1 associates with the SUMO conjugating enzyme UBC9 and promotes IRF7 SUMOylation, which promotes IRF7 stability, but negatively regulates IRF7 chromatin-binding [41,42] (Figure 3). 

## 9. Concluding Remarks

Though much has been learned about how LMP1 activates NF-κB and IRF7 pathways, important questions remain. For instance, why are LMP1 TES1 and TES2 both important for EBV-mediated B-cell transformation? What cell target genes uniquely or commonly respond to LMP1-mediated IRF7, canonical or non-canonical NF-κB activation? Which NF-κB TFs are most important for the growth and survival of LMP1-positive epithelial *versus* B-cell malignancies? Rapidly advancing next-generation nucleic acid sequencing and ChIP-Seq technologies will enable increasingly precise characterization of these questions, both in model systems and importantly, in human EBV-associated malignancies. Systematic genetic analysis of LMP1-mediated non-canonical and IRF7-pathway activation may reveal novel pathway components specific to LMP1. Increasingly precise understanding of LMP1 NF-κB and IRF7 pathways, as well as their target genes, promises to allow identification of targets whose inhibition selectively impairs the growth and survival of EBV-transformed cells, and ultimately to guide the development of rational therapeutic agents for LMP1-associated human malignancies. 
